# Identifying differential miR and gene consensus patterns in peripheral blood of patients with cardiovascular diseases from literature data

**DOI:** 10.1186/s12872-017-0609-z

**Published:** 2017-06-30

**Authors:** Agnė Šatrauskienė, Rokas Navickas, Aleksandras Laucevičius, Heinrich J. Huber

**Affiliations:** 1 Vilnius University, Faculty of Medicine, Vilnius, Lithuania; 2 Vilnius University Hospital Santariškių Klinikos, Vilnius, Lithuania; 3 Department of Cardiovascular Sciences, KU Leuven, Leuven, Belgium; 4 Institute for Automation Engineering (IFAT), Laboratory for Systems Theory and Automatic Control, Otto-von-Guericke University Magdeburg, 39106 Magdeburg, Germany

## Abstract

**Background:**

Numerous recent studies suggest the potential of circulating MicroRNAs (miRs) in peripheral blood samples as diagnostic or prognostic markers for coronary artery disease (CAD), acute coronary syndrome (ACS) and heart failure (HF). However, literature often remains inconclusive regarding as to which markers are most indicative for which of the above diseases. This shortcoming is mainly due to the lack of a systematic analyses and absence of information on the functional pathophysiological role of these miRs and their target genes.

**Methods:**

We here provide an-easy-to-use scoring approach to investigate the likelihood of regulation of several miRs and their target genes from literature by identifying consensus patterns of regulation. We therefore have screened over 1000 articles that study mRNA markers in cardiovascular and metabolic diseases, and devised a scoring algorithm to identify consensus means for miRs and genes regulation across several studies. We then aimed to identify differential markers between CAD, ACS and HF.

**Results:**

We first identified miRs (miR-122, −126, −223, −138 and −370) as commonly regulated within a group of metabolic disease, while investigating cardiac-related pathologies (CAD, ACS, HF) revealed a decisive role of miR-1, −499, −208b, and -133a. Looking at differential markers between cardiovascular disease revealed miR-1, miR-208a and miR-133a to distinguish ACS and CAD to HF. Relating differentially expressed miRs to their putative gene targets using MirTarBase, we further identified HCN2/4 and LASP1 as potential markers of CAD and ACS, but not in HF. Likewise, BLC-2 was found oppositely regulated between CAD and HF. Interestingly, while studying overlap in target genes between CAD, ACS and HF only revealed little similarities, mapping these genes to gene ontology terms revealed a surprising similarity between CAD and ACS compared to HF.

**Conclusion:**

We conclude that our analysis using gene and miR scores allows the extraction of meaningful markers and the elucidation of differential pathological functions between cardiac diseases and provides a novel approach for literature screening for miR and gene consensus patterns. The analysis is easy to use and extendable upon further emergent literature as we provide an Excel sheet for this analysis to the community.

**Electronic supplementary material:**

The online version of this article (doi:10.1186/s12872-017-0609-z) contains supplementary material, which is available to authorized users.

## Background

With the advent of affordable sequencing and high-throughput gene expression analysis, micro RNAs (miRs), small non-coding RNA molecules of 18–22 nucleotides, and their target genes have been proposed to serve as means for early diagnosis in cardiovascular and metabolic diseases [1–3]. MiRs are ubiquitously present in the vascular tissue including their presence in monocytes, macrophages, vascular endothelial cells and smooth muscle cells, platelets and exosomes [4, 5]. They thereby regulated several fundamental processes such as differentiation, growth, proliferation and apoptosis [6, 7].

The plethora of recently published research of nucleotide and genetic markers requires a systematic and reproducible analysis of the available literature. In addition, miRs are known to regulate multiple target genes. In turn, one gene can be regulated by different miRs, which makes an assessment as to how miRs influence target gene expression cumbersome. Hence, deriving the patho-physiological from gene and their target genes requires a reductionist approach that extracts the essential features of the miRs regulation pattern in a structured and biological-reasoning driven way. Based on a previous analysis [8] we here provide a systematic approach using a simple scoring scheme to identify potential regulation patterns impinged by miRs that are relevant to metabolic and cardiovascular diseases. In particular, we want to determine which miRs are specific for metabolic and cardiovascular diseases, and whether or not disease specific miRs and their target genes can help to differentiate between Coronary Artery Disease (CAD), Acute Coronary Syndrome (ACS) and Heart Failure (HF) with respect to their pathophysiological functions.

## Methods

### Information resources, search and study selection

**Fig. 1:**
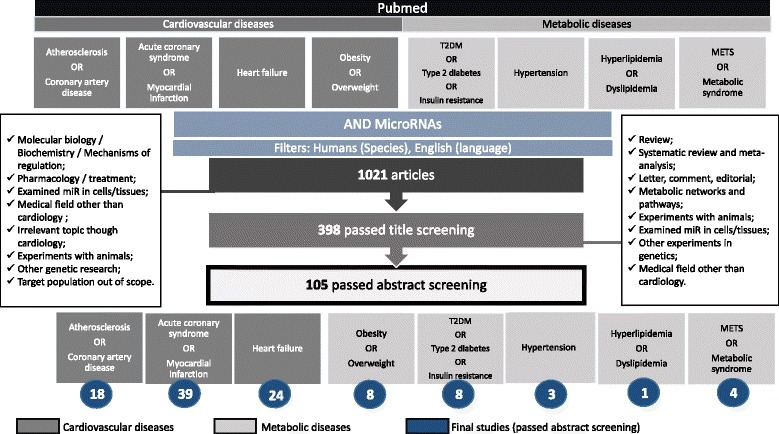
Strategy for literature search and exclusion criteria. The search strategy for retrieving published articles through the PubMed database is depicted. Thereby, the Pubmed filters “*species: human* and *language: English”* were used. The following keyword search terms were included for PubMed search: “atherosclerosis”, “coronary artery disease”, “acute coronary syndrome”, “myocardial infarction”, “heart failure”(which describes cardiovascular conditions), “obesity”, “overweight”, “T2 M”, “Type 2 diabetes”, “insulin resistance”, “hypertension”, “hyperlipidemia”, “dyslipidemia”, “METS” and “Metabolic syndrome” (which describes metabolic conditions) in conjunction to “microRNA” . The literature search yielded 1021 potentially relevant articles. Articles were excluded based on title screening using the exclusion criteria in the left box. The resulting 398 papers were then screened by reading abstract and applying exclusion criteria (right box). The remaining 105 studies were analyzed and data were extracted from each paper into an excel sheet. Diseases were grouped into cardiovascular and metabolic diseases

The Pub Med database for English-language articles was queried with respect to miRs as biomarkers in cardiovascular and metabolic disease. For cardiovascular diseases (CAD, ACS and HF) the key search term ‘(miR) AND (atherosclerosis OR cardiovascular OR acute coronary syndrome OR heart failure’ was used. In relation to metabolic diseases the search term ‘(miR) AND (biomarker) AND (overweight OR obesity OR type 2 diabetes OR insulin resistance OR hypertension OR dyslipidemia OR metabolic syndrome’ was employed. Based on abstract, we excluded experimental studies in preclinical models such as mice, rats, or pigs. We included only original research papers for the final assessment. No lower date limit was used. The strategy and outcome of our literature search is illustrated in Fig. 1.

### Scoring analysis

**Fig. 2:**
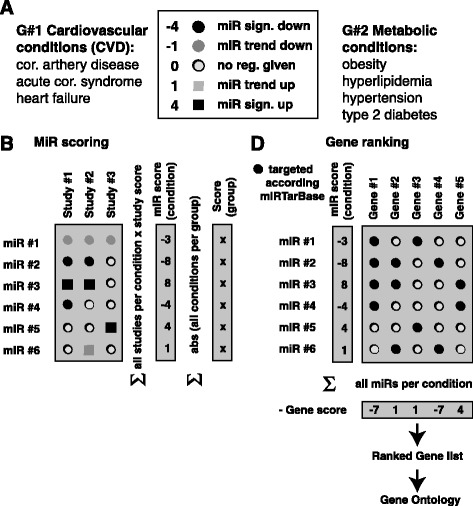
The scoring algorithm for determining most relevant miRs, genes and gene ontology terms. **a** Disease conditions were collated into a group of cardiovascular and metabolic as indicated. MiRs from literature were assigned an individual score as indicated in the box. Individual scores were dependent on whether miRs were found to be up-or down regulated, and dependent on the statistical significance of the finding as reported in literature. **b** Scores of miRs were calculated by balancing scores from each publication relevant to the condition under study. In addition, absolute values of miR scores for each condition were summarized over all disease conditions of a group, allowing us to assess its overall importance for either cardiovascular or metabolic diseases. **c** Disease specific scores from miRs were used to calculate a ranked list of target genes that are likely up-regulated or down-regulated in a certain condition. Gene-miR associations were obtained from miRTarBase, whereby only experimentally confirmed, strong relations were considered (hsa_MTI_strong). A ranking score for each gene was calculated summarizing the scores from all miRs of which the gene is a target. To correlate positive gene regulation with positive score, the sign of the score was inverted. The ranking list was then used for subsequent GO analyses using the tool Gorilla [9]

To assess the influence of miRs and their target genes in a specific disease or condition, rating scores were assigned. Our aim was thereby to include not only statistically validated markers, but also such where a trend was observed. Scoring analysis for miRs and genes was performed by Excel. Scores indicate the likelihood and direction of their regulation by assessing the overall picture in literature. For calculation of miRs scores, we first assigned a rating score for each individual miR that was identified by a particular study and in a particular condition (such as CAD, ACS or HF). This rating was set to 4 or 1 for such miRs that were found in a certain study to be significantly or non-significantly up-regulated, and −4 or −1 for such that were reported to be significantly or non-significantly down-regulated (Fig. 2a). Thereby, the absolute ratings between significant (4) and trend (1) data were designed to find an appropriate trade-off between giving credits to statistical significance, and not disregarding trends that may reveal interesting consensus patterns. We note that these factors can be easily adapted in our Excel analysis sheet that we provide to the community (see Additional file 1).

Scores for each miR were then summarized over all studies related to a certain disease condition, aiming to estimate an overall literature consensus regarding the role of the specific miR under this condition. Thereby, the scores were weighted with the study power in our analysis by classifying according smaller studies (<50 disease group patients, study score multiplied by 1), medium (50–100, by 2) and larger studies (>100, by 3). In addition, group scores for each miR were calculated by summarizing the absolute miR scores for each miR over all disease conditions of a specific disease group (Fig. 2b). Thereby, CAD, ACS and HF were grouped in the cardiovascular disease group, while diabetes, obesity, hyperlipidemia and hypertension were considered as metabolic diseases.

Target genes for each miR and associated to each disease were obtained querying the miRTarBase (18) that was chosen due to its widespread use and extensiveness. Only experimentally (qRT-PCR, Luciferase assays, Western Blots) confirmed strong mRNA- miRs interactions were considered (qualifier “hsa_MTI_strong”). Similar to miRs, genes were assigned a ranking score by summarizing the scores of all miRs of which it is a target. Positive to negative scores were used to rank genes from the direction of the most likely down regulated to the most likely up regulated. Therefore, the sum of miR scores was multiplied by −1 to account for the fact that miR and target gene regulations are negatively correlated. Overall importance for miRs and gene score was assessed for the entire cardiovascular disease group. MiR and gene scores were further ranked according their overall importance.

The identified ranked gene lists for each disease condition were subsequently subjected to a gene ontology (GO) analysis using the tool GORILLA (http://cbl-gorilla.cs.technion.ac.il/) [9]. Thereby, only genes with an absolute score of −6 were chosen, meaning that these genes were associated to at least one miR that had a score of +/− 6, or to an adequate combination of miRs with lower scores. For the GO analysis an enrichment cut off of E-3 was used in the tool and default parameters for ranked lists and for calculating GO functions were used. This led to the identification of enriched pathways, characterized by a gene ontology (GO) term for CAD, ACS and HF. Commonality of genes and GO terms between the conditions were analyzed and plotted as VENN-diagram.

The Excel sheet used for analysis including a detailed description of its usage is provided in the supplement (Additional file 1: Methods and Table).

## Results

### Scoring reveals distinct miR markers in metabolic and cardiovascular disease

To associate miRs found in peripheral blood with specific metabolic and cardiovascular diseases, we have first carefully screened literature and subsequently developed a scoring mechanisms to extract meaningful consensus patterns (Fig. 2, Methods, and Additional file 1: data 1). As indicated in Fig. 2a, we have thereby assigned scores per miR and per identified study depending on the significance of the miR. Then, we assigned a positive and negative sign to miR scores depending on whether they were found to be up and down regulated. We finally summarized miR regulation scores over different studies that first related to a certain disease condition and subsequently summarized scores over all disease conditions per group (e.g. the cardiovascular disease group consisting of CAD, ACS and HF).

**Fig. 3:**
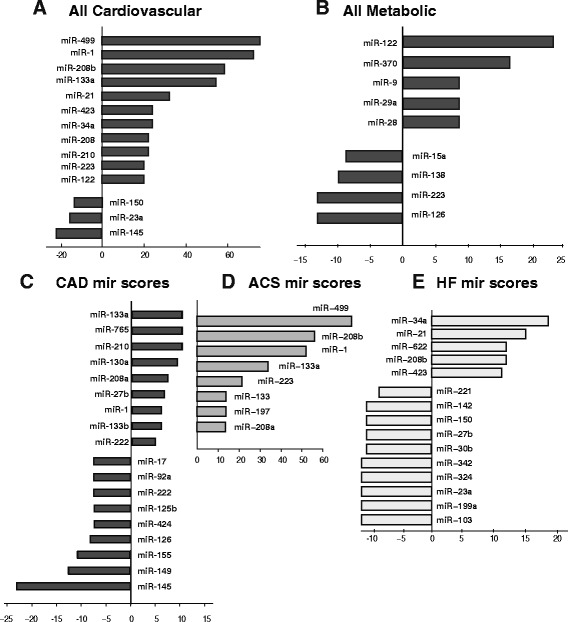
Scoring analysis of miRs and their target genes in cardiovascular and metabolic diseases following the procedure of Fig. 2. **a-b** MiR were scored and scores were ranked from highest to lowest on a disease group level for all cardiovascular (**a**) and metabolic diseases (**b**). Strikingly, with exception of miR-122, which was found upregulated (positive score), and −223 which was counter regulated between both disease groups, no overlap in the depicted miRs was found between both disease groups, allowing for differential markers. **c-e** MiR scores were calculated for (**c**) Coronary Artery Disease, (**d**) Acute Coronary Syndrome and (**e**) Heart Failure following the procedure of Fig. 2 and ranked according an overall importance from top till bottom as stated in the text. The highest positive and negative scores for each condition were given. While scores for CAD and were found to be more equally distributed between positive and negative numbers, miRs related to ACS had mainly positive scores, indicating a general up-regulation of the miR transcriptome. Analysing miR scores between the three above three conditions revealed miR-1, miR-208a and miR-133a to distinguish ACS and CAD form HF

Applying this scoring scheme, we were first interested in identifying which miRs can be considered as distinguishing markers between cardiovascular and metabolic disease conditions. We therefore grouped coronary artery disease (CAD), acute coronary syndrome (ACD), heart failure (F) into the first group while lumping obesity, hyperlipidemia, hypertension and type 2 diabetes into the second. We found that in the group of cardiovascular diseases miR-1, −499, −208b, and -133a were mostly decisively upregulated (Fig. 3a), while miR-145, −23a and −150 were markedly downregulated. In contrast, in the group of metabolic diseases, miR-122 and -370 were decisively upregulated, while miR-126, −223 and −138 were scored to be largely downregulated. Strikingly, no high scoring miRs were found to be common in both disease groups, indicating that both pathology groups were affected by a post-transcriptional program based on distinct miRs.

### Differential miR analysis to distinguish coronary artery disease, acute coronary syndrome and heart failure

As precise disease markers are necessary to study potential progression from coronary artery disease to acute coronary symptom to heart failure, we looked for miRs that are common or differential to each of the cardiovascular diseases (Fig. 3 c- e). We found that in the case of coronary artery disease (CAD), miR-133a −765 were most decisively up- and miR-145 and -149 downregulated. Interestingly, in acute coronary syndrome (ACS), only miRs with positive score were observed, indicating upregulation, whereby miR-499, −1 and those of the −208 group were the most prominent one. In contrast, in heart failure (HF), positively and negatively regulated miRs were identified. Additionally, miR-1 together with miRs of the −208 and −133 family were found co-regulated in CAD and ACS, but not to be decisively involved in HF in metabolic disease, providing their potential as differential marker for ACS and CAD.

### MiR target genes as differential marker between cardiovascular diseases

We next investigated the targeted genes of the most decisively regulated miRs to establish genetic markers that may identify of distinguish between CAD, ACS and HF. We therefore employed the association between miRs and their target genes form the miRTARbase database [10]. We then assigned gene scores, whereby we summarized over all scores of their parental miRs that were associated to a specific disease. We finally accounted for the negative relation between miR and gene regulation by altering the sign of the resulting scores.

**Fig. 4:**
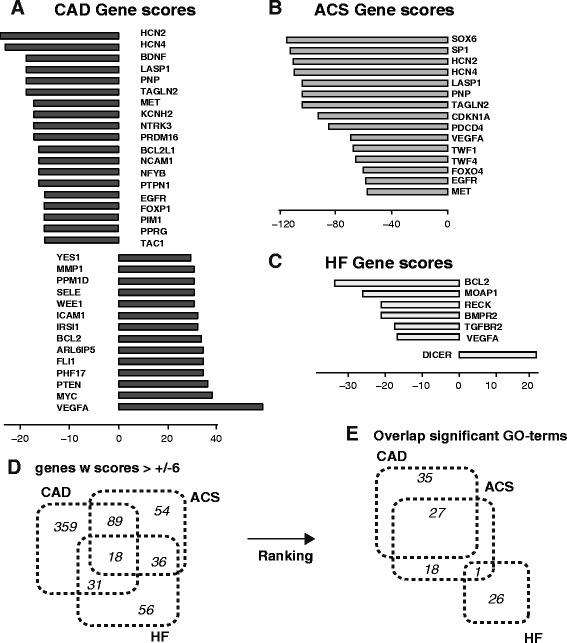
Influence of miR regulation on target genes and predicted consequences on pathophysiological function. **a-c** Gene scores were calculated for (**a**) Coronary Artery Disease (**b**) Acute Coronary Syndrome and (**c**) Heart Failure following the procedure of Fig. 2. The highest positive and negative scores were given for each. Positive and negative genes scores indicate the likelihood of gene up or down-regulation, respectively. Following the patterns of miRs from Fig. 3c-e, genes scores related to CAD and HF were more evenly distributed between positive and negative values than such to ACS. HCN2/4, LASP1 and EGFR were negatively regulated in CAD and ACS, while not being found decisively regulated in HF. The anti-apoptotic protein BLC-2 was found oppositely regulated between CAD and HF, while no relevant score was identified in ACS. Different isoforms of the Forkhead transcription factor family (FOXO) were identified in CAD (FOXO3 with score 28 not shown and FOXP1) and ACS (FOXO4). Finally, VEGF-A upregulation was found as unique marker of CAD, SOX, SP1 as unique markers of ACS, and MOAP1 and DICER as markers of HF. **d-e** Venn diagrams present the overlap of (**d**) genes, and (E) gene ontology terms. Gene ontology terms were derived from an analysis of ranked gene lists using the tool Gorilla [9] and exploiting our scoring algorithm, whereby only genes with absolute scores greater or equal 6 were taken into account to avoid bias due to unspecific gene regulation. While overlap of considered genes between all conditions were by a similar amount (overlap of all are similar on a percentage scale), the GO analysis using ranked lists indicated a much higher overlap between CAD and ACS, while GO terms for heart failure were nearly distinct to both others. Results indicated that our ranking procedure provides additional information content to allow a functional assessment and a differential analysis of disease function

Not surprisingly, the pattern of consistently downregulated miRs in ACS resulted in mostly positively scoring genes (Fig. 4b), suggesting a robust genetic upregulation. By similar means, the relatively equal distribution between positive and negative scores in miRs was preserved on a gene level for CAD (Fig. 4a), and to a lower extend for HF (Fig. 4c). As differential gene marker, we found HCN2, HCN4, LASP1 and the endothelial growth factor receptor EGRF as commonly regulated in CAD and ACS, but not involved in HF. In contrast, BDNF was regulated in all disease (albeit at lower score − 58 in ACS and −13 in HF, and hence not shown), yet the precise role of this neurotrophic factor in cardiovascular pathophysiology remains elusive. As further differential marker, the apoptosis-protective gene BCL2 was found to be upregulated in CAD, not regulated in ACS and downregulated in HF, the latter indicating a reduced protection against apoptosis in HF. Likewise, we note that vascular endothelial growth factor A (VEGF-A) was upregulated in CAD and downregulated in ACS and HF In contrast, we found thatthe proto-oncogene MYC may be a specific genetic markers for CAD, while the hematopoietic transcription factors SP1 and SOX6 are potential specific markers for ACS. Interestingly, the ribonuclease DICER that is essential for small RNA processing was found to be likely upregulated during HF, suggesting a potential feedback of this miR target gene to further miR processing.

### Analyzing overlap between ACS, CAD and HF on a gene expression and on a GO level

We finally aimed to investigate overlap of genes and functions between the three diseases. We therefore analysed all genes with absolute scores greater than 6 and grouped them in a VENN diagrammed according to their disease. However, looking at the amount of disease common genes revealed percentagewise a relatively equal overlap between all diseases, making it impossible to distinguish CAD from ACS and HF by patterns of gene regulation (Fig. 4d). We thus were wondering if a gene to function mapping would reveal a higher similarity between two diseases compared to the other. We therefore performed a gene ontology analysis of a ranked list of genes of each disease ranking from the most negative to the most positive score and determined GO functions by the tool *GORILLA*. Most interestingly, we found a good overlap of GO functions between CAD and ACS with 27 functions. In contrast, only 1 GO functionoverlapped between ACS and HF and even no overlap between CAD and HF was detected (GO enrichment level E-3). We therefore conclude that gene to function mapping may reveal hidden similarities between CAD and ACS compared to HF in peripheral blood, which are not detectable on a miR or gene expression levels.

## Discussion

### Relevance and interpretation of the scoring mechanisms

Consensus patterns of circulating miRs in blood and body fluids are currently gaining wide interest in diagnostic research for cardiovascular and metabolic diseases, cancer and neuro-pathologies [2, 11, 12]. We have therefore provided a systematic analysis of literature data from miR biomarkers detected in peripheral blood and employed a simple scoring algorithm to identify consensus patterns of miRs and their target genes. MiR scores were weighted according their statistical relevance and the study size, and added over the identified literature per disease condition and disease group. The sign of the miR and gene scores can hence be interpreted as regulatory profile (up- or downregulation) and their absolute values as average likelihood to be involved in a certain disease. Likewise, as miRs with higher relative expression in diseases are more likely to be identified by clinical studies, the absolute value of miRs and their target genes can alternatively be considered as potential surrogate for their differential expression under a certain disease condition. We nevertheless note that, following the philosophy to keep the analysis simple, the size of the study groups was only considered by a simple classification scheme. We therefore will subsequently discuss our findings in the context of large-scale studies.

### Potential differential miR markers of and their relevance in metabolic and cardiovascular diseases

Our analysis revealed miR-122 as the only commonly regulated marker in cardiovascular and metabolic diseases, while miR-223 was counter-regulated between both disease classes. Indeed miR-122 was mentioned in 10 analyzed studies including three major large-scale studies. As such, Li et al. [13] examined 117 acute myocardial infarction, 287 unstable angina, 81 stable angina patients, 72 high-risk individuals and 16 healthy controls and proposed miR-122 as biomarker of ACS. In contrast, the study of Gao et al. [14] investigated 255 subjects with hyperlipidemia and 100 healthy controls and found out that miR-122 is upregulated in patients with hyperlipidemia and associated with coronary artery disease. Moreover, Wang et al. [15] investigated 56 obese subjects and 56 healthy controls and found out that circulating miR-122 is associated with obesity and insulin resistance in young adults. The findings of these three large studies together with the relative high scoring in both disease groups (with the scoring not explicitly considering study size) confidently suggest involvement of miR-122 in cardiovascular and in metabolic diseases.

We further have identified miR-1 as the second most highly ranked miR in all cardiovascular diseases and our results suggest is at differential marker to distinguish CAD and ACS from HF. Indeed, miR-1 was the most frequently reported miR in our analysis set with overall 19 studies. As such, Ai et al. [16] investigated 93 patients with acute myocardial infarction and 66 healthy controls and found out that miR-1 is upregulated in patients with acute myocardial infarction. Furthermore, the study of Zhang [17] stated that miR-1 is not only a biomarker for AMI, but also predicts heart failure after acute myocardial infarction. Consistent with our scoring scheme, miR-1 as diagnostic and prognostic biomarker was mentioned in CAD and ACS, but was not found in HF.

Our study further proposed members of miR-families miR-133 and -208 as upregulated in CAD and ACS. Indeed, three major studies [18], Peng et al. [19] and Eitel et al. [20]) confirmed the upregulation of miR-133a in patients with acute coronary syndrome. Likewise, the miR-208b was mentioned in 10 studies. Specifically, this miR was increased in acute myocardial infarction patients with left ventricular remodeling [21] and found upregulated in 1155 unselected patients with acute chest pain [22].

Finally, our analysis identified miR-499 as highest scored miR in the cardiovascular disease group in general, and in the ACS subgroup in particular. Indeed, miR-499 was mentioned in final 16 studies and found significantly upregulated in patients with acute chest pain in the large-scale study of [22] and in patients with AMI ([23, 24]). Moreover, Gidloef [25] found out that miR-499 is not only biomarker for AMI, but is also associated with long-term prognosis following myocardial infarction.

### Relevance of genetic markers

We subsequently extended the scoring method to gene scores using the relation between miRs and their target genes as obtained MirTarBase. We nevertheless note that only genes were considered that result from differentially regulated miRs in blood and that other gene regulatory information that may overlay the detected gene expression profile was not considered. Nevertheless, the following gene regulatory information was identified in our analysis.

Our analysis identified the proteins of the pacemaker channel HCN2 and HCN4 as regulated marker in CAD and ACS. The proposed downregulation in CAD and ACS was due to the upregulation of miR-1 and the miR-133 family in both diseases. Interestingly, currently little research has been performed that relates HCN2/4 function to a functional role in both diseases, suggesting potential for their investigation. In turn, no miR with target genes HCN2 or HCN4 was identified in HF according to the studies selected by our criteria. Despite this absence of miR-dependent regulation of HCN2/4 in heart failure, some mechanistic role of HCN2/4 has been nonetheless attributed to hypertrophy and tachycardia. As such, mouse studies suggested increased expression of HCN2/4 to prolonged ventricular repolarization in hypertrophic cardiomyocytes, thereby diminishing repolarization reserve [26]. Moreover, mutations in HCN4 were associated with familial inappropriate sinus tachycardia [13], but the role of deregulated expression during disease onset remains undefined.

Our study further proposed reduced BDNF (Brain-derived Neurotrophic factor) levels as relevant to ACS and to a minor extent indicative for CAS and HF. This was due to upregulation of miR-1 in ACS, upregulation of miR-1 and miR-210 in CAD, and of miR-1, miR-22 and miR-210 in HF. Consistent with our results, Takashio et al. [27] found low plasma serum levels reduced in a study of 58 patients with HF compared to healthy control and decrease BDFN was associated with HF severity. Likewise, Kadowaki et al. found serum BDNF lower in 134 CHF patients than in 23 controls [28]. Moreover, serum BDNF was also lower in patients with cardiac events than in event-free patients. In contrast our results, Hang and colleagues found BDNF significantly enhanced in ACS in rats and patients and BDFN treatment markedly reduced infarct size in rats [29], while no role of BDNF in CAD pathogenesis has been described comprehensively yet.

A further growth factor, vascular endothelial growth factor A, VEGF-A, has been found to have high positive scores in CAD, indicating a very likely upregulation and hence a potential increase in neo-angiogenesis. This high score was a result of the disease specific downregulation of the highly negatively scoring miR-145 together with regulation of several other miRs (−15b, −16, −17, 20a, 20b, 21, −29b, −34a, −126, 133a, −150 and −195). Indeed, increased neo-angiogenesis has been positively correlated with atherosclerotic plaque formation [30, 31]. In contrast, VEGF-A was negatively scored in ACS and more prominently in HF, suggesting attenuation of angiogenesis under more acute and remodeling conditions. Indeed, disrupted angiogenesis is considered as contributing factor to the transition to HF [32].

Since BCL-2 is a well-known anti-apoptotic protein, BCL-2 regulation in cardiac disease may be instrumental to react to or compensate for inflammatory or oxidative stress [33]. Indeed, we found BCL-2 to be elevated in CAD due to the downregulation of several parental miRs (miR-156, −16, 20a, −21, −29ab, −143, −181d, −195). The involvement of many miRs in regulating CAD therefore suggest a broad transcriptional program involved in its regulation, presumably due to its high relevance in a terminal process such as apoptosis. Despite this broad regulation, however, the role of BCL2 in CAD seems underexploited in literature. Additionally, BCL-2 was found downregulated in HF due to upregulation of miR-21, miR-24a and miR-200b, thereby suggesting elevated apoptosis susceptibility. Indeed, BCL2 is involved in myocyte cell loss that contributes to a variety of cardiac pathologies, including heart failure [34]. However, the exact role of BCL2 and its agonistic and antagonistic family members in literature remains elusive [35]. As such, Latif and colleagues [36] found the pro-apoptotic BCL2 family members BAX and BAK upregulated in patients with heart failure. Nevertheless, in contrast to our study their study also found BCL-2 and a further anti-apoptotic protein, BCL-XL as upregulated, suggesting not only elevated apoptosis in HF, but also the presence of a possible concomitant, compensatory anti-apoptotic mechanism. Finally, the role of BCL2 in ACS was found negligible in our study (score 13) which is in line with the absence of identified studies in literature.

Our study suggested the hematopoietic transcription factor SP1 to be highly negatively scored and hence downregulated in ACS. This was due to the upregulation of their parental miR-1 and miR-133/−133a, which scored highly positive. Importantly, SP1 is a decisive transcription factor of endothelial nitric oxide synthases (eNOS), a key anti-oxidant in the vascular endothelium. It is therefore likely that SP1 depletion can alter the endothelia’s capacity to cope with oxidative stress and hence be an indicator of cardiac risk under ischemic circumstances. To this end, Xu et al. suggested that SP1 is involved in the induction of Cox-2 in hypoxic human umbilical vein endothelial cells, further linking vascular hypoxia to SP1 expression [37]. By similar means, it was shown that SP1 is regulated by Insulin-like growth factor IGF-1 signaling, thereby further linking SP1 downregulation to ischemic conditions that cause deprivation of trophic factors [38]. Finally, a large-scale clinical study has revealed that mutations in the SP1 binding site of the promoter in ABCG1 reduced SP1 binding and increased risk of myocardial infarction and ischemic heart disease [39].

SOX6 was identified in our study as the gene with the highest negative scores in ACS, suggesting a high likelihood of its downregulation. SOX6 is a transcription factor and important regulator of cardiomyocyte development, acting in the BMP pathway of cardiac differentiation [40]. Decreased SOX6 expression may hence be linked to a de-differentiated and hypertrophic phenotype, decreasing cardiac functionality and increasing likelihood of ACS and HF. Consistent with the notion of decreased SOX6 and cardiac muscle impairment, SOX6 expression has been positively associated with myofiber-specific gene expression and muscle performance [41].

CDKN1A and MYC are important regulators of cell cycle and differentiation. While our study suggest CDKN1A downregulation in ACS and a MYC downregulation in ACS, direct literature evidence on these genes rather suggest a positive role in hypertrophy and HF. Indeed, MYC was found to be upregulated in the adult myocardium in response to a pleiotropic range of hypertrophic stimuli [42, 43].

Finally, DICER was found as the gene with the most negative score in HF. Indeed, DICER deletion was linked to dilated cardiomyopathy in mutant mice [44]. DICER is decisively involve in regulating short RNAs and, hence, in regulating the miR-based post-transcriptional program itself. Therefore, DICER is likely to have more pleiotropic effects governing hypertrophy and HF.

### Relevance of GO mapping with respect to disease similarities

Finally, studying study genes in their combination by a gene ontology analysis revealed a closer relation between CAD and ACS than both diseases had to HF. Indeed, we expect that the transcriptional program that regulates miR-expression within certain diseases cannot be decoded on a single gene level, but requires an analysis of gene expressions in their interrelation. Arguably, CAD and ACS show phenomenological similarities from a cardiology point of view compared to heart failure. From their essence, CAD and ACS are regarded as inflammatory diseases of peripheral or coronary vessels, respectively [45, 46]. Both, CAD and ACS are associated with coronary atherosclerosis progression, whereby CAD often precedes ACS [47]. In contrast, HF can be caused by a variety of abnormalities, including pressure and volume overload, loss of cardiac muscle, primary muscle disease or excessive peripheral demands such as high output failure. In the usual form of HF, the heart muscle has reduced contractility. Besides these essential and etiological features, CAD and ACS differ from HF by their main symptoms. Specifically, while both vessel diseases cause pain and discomfort in the heart area of limited duration and suddenness, HF presents itself by dyspnoea, swelling and fatigue.

From a disease progression perspective, atherosclerosis/CAD often progresses into ACS, whereby the atherosclerotic plaque mass may bulge into the lumen and cause a haemodynamic obstruction and angina pectoris symptoms [47]. Morever, clinical trials of post-MI patients suggest that prompt and appropriately targeted therapy can lower the risk of development of ventricular dysfunction and overt heart failure after ischemic injury, thereby further suggesting progression from stable (CAD) to unstable (ACS) vessel disease [48, 49]. Nevertheless, HF also often follows coronary heart disease and in fact accounted for 67% of congestive heart failure cases in the 1980s according to the Framingham heart study [50]. Predictions as to whether CAD more likely progresses to ACS or both to HF are therefore difficult, and hence the difference in the GO term list between ACS/CAD and HF cannot be solely explained from a perspective of disease progression.

Finally, in contrast to CAD and ACS, HF is an end-stage disease and a clinical syndrome that can result from a wide variety of primary causes and which involves many organs outside the heart such as the kidney [51]. The HF clinical syndrome is further aggravated by the presence of a plethora of comorbidities such as sleep apnoea [52], diabetes type II and atrial fibrillation [51]. Hence, these pleiotropic causes and co-morbidities could account for the distinct gene ontology features that were proposed by our study and hence give rise to a distinct set of diagnostic markers that can be determined from peripheral blood [53].

### Limitations of the study design

The philosophy of our study design was to provide a simple approach to assess the common relevance of a broad range of studies of miR biomarkers. Our approach is hence complementary to classical meta-analyses that require comparability of statistical analyses. Yet, due to considerable variations in the methodology of primary clinical studies, such meta-analyses hence only allow addressing a much smaller subset of comparable studies, and therefore are often not able to extract broad conclusions. While our approach does not provide a similar statistical rigor, it allows us to address a broader range of studies. We thereby can also consider cues that emerge from several studies, which each by themselves would not have enough statistical power, but in their combination may reveal interesting trends for subsequent studies.

Likewise, besides having defined inclusion criteria of studies as outlined in Fig. 1, we refrained from using measures to assess overall study quality, as literature consensus how to best assess miR study quality is not yet reached. Indeed some co-authors of this study recently commented on the need of unifying standardisation/ normalization of reference miRs, adjustment for comorbidities and medication, and implementation of gold standards for data acquisition and planning across clinical studies for circulating miRs [8]. Nevertheless, the provided Excel sheet is flexible enough that these quality measures can be chosen and applied by other researchers in potential follow-up studies according to their own criteria.

Finally, all approaches in secondary literature, including our consensus approach, rigorous meta analyses and classical review paper are always prone to an inherent bias towards ab initio marker-selection in primary studies. Specifically, we cannot exclude the possibility of a trend in literature for investigating ‘confident’ markers to minimize study risks (especially when designing large-scale studies), analyzing markers that are in the focus of the respective research groups or of affiliated groups, studies designed by following leaders in the field, or such studies motivated by needs of the pharmaceutic industry [54, 55].

## Conclusion

We provided an analysis for identifying consensus patterns on genes and miRs found in literature studies of peripheral blood related to several metabolic and cardiovascular diseases. Our analysis revealed that metabolic and cardiovascular disease are largely characterised by a different miR-based post-transcriptional program and identified differential markers for ACS, CAD and HF. Performing a gene ontology revealed a surprising resemblance between CAD and ACS compared to HF, arguing for a shift from a single gene-centred to a functionality-based and holistic view on literature data. Our analysis is easy to use and can be easily extended for including future studies by an Excel-sheet we provide to the community (Additional file 1: Table and text).

## Additional file

Additional file 1: Supplementary Information 1: Structure of the Analysis Excel sheet. (ZIP 3155 kb)
