# Supplementary material for: Identifying differential miR and gene consensus patterns in peripheral blood of patients with cardiovascular diseases from literature data
Source: BMC Cardiovasc Disord. 2017 Jun 30;17:173. doi: 10.1186/s12872-017-0609-z (PMC5493858; doi:10.1186/s12872-017-0609-z)
Supplement: Additional file 1: — Supplementary Information 1: Structure of the Analysis Excel sheet. (ZIP 3155 kb) [file 12872_2017_609_MOESM1_ESM.zip › 12872_2017_609_MOESM1_ESM/Supplement 1_Satrauskiene_revision et alR1.docx]

**Supplementary Information 1: Structure of the Analysis Excel sheet**

**General information:**

The Excel sheet contains a control sheet, “primary data sheets” for the miRs related to each disease condition (CAD, ACS, HF, diabetes, obesity, hypertension (htens), hyperlipidemia (hlip) and metabolic syndrome (met)), “secondary data sheets” with the genes that are associated to these miRs (CAD_gene, ACS_ gene, etc.), and “tertiary data sheets” where the scoring of miRs and genes per disease, group and between groups is performed (analysis_mir_score, analysis_gene_score). The sheet ‘hsa_MTI_strong’ was extracted from MirTarbase linking genes to their parent miRs. Finally, the data sheets ‘genes final analysis’ and ‘GO’ contain the calculations for common genes and GO terms in the Venn diagrams in Fig 4D and E, respectively.

**Structure of the Control Sheet:**

In cells C1 and C2 of the control sheet, the scores that shall be associated to non-significant and significant genes in the primary data sheets are defined and set to 1 and 4, respectively. Further, if an exclusive list of important miRs (E2-E12) shall be considered, the flag in C4 has to be set to zero, although this feature was not used in current analyses. In turn, if certain miRs shall not be considered, a ‘blacklist’ has to be assigned from M2 downwards with the number of blacklisted genes specified in N2. This feature was intended to collect miRs and their target genes that come from a non-specific ‘metabolic background’ and subtract their influence from all cardiac conditions (CAD/ACS/HF). The feature is switched off by setting N2 to 1 and to replace the first miR in M2 by ‘dummy’. If wished, such metabolic background miRs can be obtained from those are highly ranked in column M of ‘analysis_mir_score’, Finally, the drop-down menu in B7 can specify from which sources the miRs are taken, such as all (for taking into account all sources), all serum or all plasma. The selection of all these control parameters influences all primary and secondary sheets.

**Structure of the primary data sheets (miR data collection)**

The primary data sheets are those from which miR-scores for each disease are calculated from literature evidence. The sheet names ‘CAD’, ‘ACS’, ‘HF’, ’diabetes’ , ‘obesity’, ‘htens’, ’hlip’, ‘met’, thereby describe the calculations for coronary artery disease, acute coronary syndrome, heart failure, diabetes, obesity, hypertension, hyperlipidemia and metabolic syndrome, respectively. The sheets contains the name of the miRs from literature that are associated to the respective condition (column A). Column (B) includes their significance (level p=0.05; y=yes or n=no) and column C their regulation profile (+ or – for up or down). Further, columns (D-I) given informative text which is not subjected to calculation. An individual study score for each miR per condition and study is calculated (column J) using the weight for significance and non-significance according to the cells C1 and C2 of the control sheet.

MiR scores obtained per study are also given column J, whereby these scores may be optionally modified through flags 0 or 1 in column P and Q, although both options were not chosen in the current analysis and, hence, the flags were all set to 1. The first option (flag in column N) was foreseen to allow the user to perform the analysis only for miRs detected within a specific source (e.g. plasma, serum or all), whereby the settings from the control sheet (cell B7) was chosen (default =’all’). The second option allows to define a ’blacklist’ of miRs that will not be included into the scoring and that is defined in the control sheet (cell M2-M7 for miRs and N2 for the number of blacklist miRs). Finally, disease scores for miRs are obtained by multiplying the particular miR study score by the study power (column K with power score = 1 for studies <50 disease group mpatients, score=2 for medium size (50-100) and 3 for large size) and summarising each miR over all studies and are calculated in the large matrices starting with column S. Results of those scores are calculated as sums from these matrices, transferred to columns N and O and are used for further calculations of the tertiary (analysis) sheets.

**Structure of the secondary data sheets (gene data collection)**

The secondary data sheets calculate the gene scores per disease, availing of input from the miR scores from the primary sheets and using the algorithm described in the methods above.

The miR-gene relations were taken from “miRTarBase”, whereby an excerpt for human miRs and validated strong interactions was taken and stored in the sheet “hsa_MTI_strong”. From this data base, miR-gene relations were obtained for each disease in given in columns of the secondary sheet (A-E). These include the name of the miR from the primary tables in column A, their index in column B, the miR name with which it was associated in “hsa_MTI_strong.csv” in column C and table index of this miR in “hsa_MTI_strong” in column D. The genes identified from this interaction were given in column E. An alphabetic and non-redundant list of the genes was given in column G. The number of target genes per miR was given in column H, and an accumulative list of column H is given in column E. This accumulative list is used for technical calculations in the items of the matrix at the right hand side starting with Q. Thereby, indirect addressing is used to check if a specific gene (columns of matrix) is associated to a specific miR (defined in the rows) and if so, the miR score is copied into a specific matrix element. Results of the scoring per individual condition are given by columns (K) and (L) which are used for further analysis.

**Structure of the tertiary data sheets (miR and gene summary over conditions)**

In ‘analysis_mir_score’ and ‘analysis_gene_score’ the miR and gene scores, respectively, are collated for all conditions (A-I) and a summary score for cardiovascular (column J) and metabolic conditions (column K) is calculated. A summary of absolute scores for both conditions is given as this allows characterization of the general impact of these miRs (L) per disease group (M; the latter one to assess the background). To avoid a bias given by the different amount of studies per condition, scores in each condition are also calculated in percentage to maximum absolute score for the respective condition (P-S respectively). All other data and graphs are used for data storage only and are irrelevant to the calculation.

**Other Excel Worksheets**

The sheet ‘has_MTI_strong’ is the excerpt from the ‘MiRTarBase’ database that was used for our analysis. Finally, the sheet ‘compare_revision’ depicts the differences of the analyses before and after revision. Specifically, as suggested by the reviewer, a scoring scheme was used for assessing study size. This scoring scheme is introduced in columns ‘M’ of the primary (miR) data sheets and can be switched off, by setting all values of the column in each data sheet to zero.
